# miR-550a-3-5p acts as a tumor suppressor and reverses BRAF inhibitor resistance through the direct targeting of YAP

**DOI:** 10.1038/s41419-018-0698-3

**Published:** 2018-05-29

**Authors:** Min Ho Choe, Yina Yoon, Joon Kim, Sang-Gu Hwang, Young-Hoon Han, Jae-Sung Kim

**Affiliations:** 1 Division of Radiation Cancer Research, Korea Institute of Radiological and Medical Sciences, Seoul, 139-706 Korea; 2 Laboratory of Biochemistry, Division of Life Sciences, Korea University, Seoul, 02841 Korea; 3 Radiological and Medico-Oncological Sciences, University of Science and Technology, Daejeon, Korea

## Abstract

Although evidence has emerged to suggest that YAP overexpression is a crucial factor for tumor progression and resistance to targeted drugs in multiple cancers, the miRNA-mediated YAP regulation is still unclear. Here we show that the novel miR-550a-3-5p acts as a tumor suppressor and reverses BRAF inhibitor resistance through the direct targeting of YAP. Our data showed that the miR-550a-3-5p suppressed cell proliferation, metastasis, and tumor sphere formation through the direct inhibition of YAP and its oncogenic pathway in various cancer cell types. In addition, we showed that the YAP signature was associated with poor survival of colon cancer and identified an inverse correlation between miR-550a-3-5p and YAP in colon cancer tissues. Interestingly, this inverse correlation was regulated in a density-dependent manner. Furthermore, high levels of miR-550a-3-5p were associated with a good prognosis of esophageal cancer, which was suggestive of the clinical relevance of miR-550a-3-5p-mediated YAP regulation in multiple cancers. Importantly, we demonstrated that miR-550a-3-5p treatment sensitized vemurafenib-resistant colon and melanoma cells through YAP inhibition with reduced AKT activity. Moreover, the tumor-suppressive activity of miR-550a-3-5p and its sensitization effect for vemurafenib resistance were also observed in tumor xenograft models. Collectively, our data suggest that miR-550a-3-5p acts as a tumor suppressor through the targeting of oncogenic YAP and may be a new therapeutic tool for YAP-mediated BRAF inhibitor resistance in BRAF-mutant cancer cells.

## Introduction

Yes-associated protein (YAP; also known as YAP1 or YAP65), a transcriptional co-activator, has recently emerged as a critical oncogene in multiple cancers. YAP is a key downstream effector of the Hippo signaling pathway, which controls organ size, development, and tumorigenesis through the modulation of cell proliferation and apoptosis^1,2^, and is tightly regulated by upstream kinases and their adaptors, such as Mst1/2, Sav1, and Lats1/2, which exerts tumor suppressive activity in several cancers^1,2^. The phosphorylation of YAP leads to its ubiquitination, degradation, and cytoplasmic retention, whereas de-phosphorylated YAP, by the inactivation of the Hippo pathway, is translocated into the nucleus and activates various target genes, such as connective tissue growth factor (CTGF) and cysteine-rich angiogenic inducer 61 (CYR61)^1,2^. YAP-driven transcriptional activation promotes various oncogenic properties, including cell proliferation, anti-apoptosis, and cancer stemness^1,2^.

YAP overexpression is closely associated with resistance to anticancer therapy in various cancer models^3^. Recent studies have indicated that YAP overexpression can substitute functionally for the inhibition of oncogenic KRAS activity^4^. In addition, two groups independently reported that YAP overexpression confers BRAF inhibitor resistance in BRAF-mutant melanoma and non-small cell lung cancer (NSCLC)^5,6^, which suggested that YAP inhibition could overcome BRAF inhibitor resistance in BRAF-mutant cancer cells. Although YAP overexpression is a critical factor for tumor progression and resistance in multiple cancers^2,3^, genetic alterations in Hippo-YAP pathway components are rare^1^. Thus, it has been suggested that YAP overexpression and activation might be associated with other oncogenic drivers or epigenetic regulation^1^. However, the regulatory mechanisms of YAP overexpression in multiple cancers are still unclear.

MicroRNAs (miRNAs), small non-coding RNAs of ~19–25 nucleotides, suppress gene expression by binding to complementary sequences in the 3′ untranslated region (UTR) of mRNAs to control various biological process, including survival, apoptosis, cell cycle, and gene regulation^7^. Dysregulated miRNAs play critical roles in tumor progression by acting as an oncogene or tumor suppressor in human cancers^7^. Thus, the potential applications of miRNAs for the clinical uses of cancer monitoring and therapy are an emerging topic in the field of anticancer treatment. Recently, several studies indicated that miRNAs were also important in the development of tumor resistance to various anticancer drugs through the regulation of the resistance-associated signaling pathways^8,9^. For example, tumor resistance to EGFR and MET receptor tyrosine kinase inhibitor or TRAIL are closely associated with specific miRNAs in NSCLC or liver cancer^10,11^. Although few miRNAs associated with BRAF inhibitor resistance have been reported^12,13^, there are still many unknown regulatory miRNAs for YAP-mediated BRAF inhibitor resistance.

In the present study, we showed that novel miR-550a-3-5p directly suppressed oncogenic YAP and exerted tumor-suppressive activity in various cancer cells. In addition, we demonstrated that miR-550a-3-5p treatment could sensitize BRAF inhibitor-resistant colon cancer and melanoma cells. Therefore, our data provided evidence that miR-550a-3-5p acts as a tumor suppressor via YAP inhibition in multiple cancer cells and a novel therapeutic tool for BRAF inhibitor resistance in BRAF-mutant colon and melanoma cells.

## Results

### miR-550a-3-5p has tumor suppressive activity in various cancer cells

**Fig. 1:**
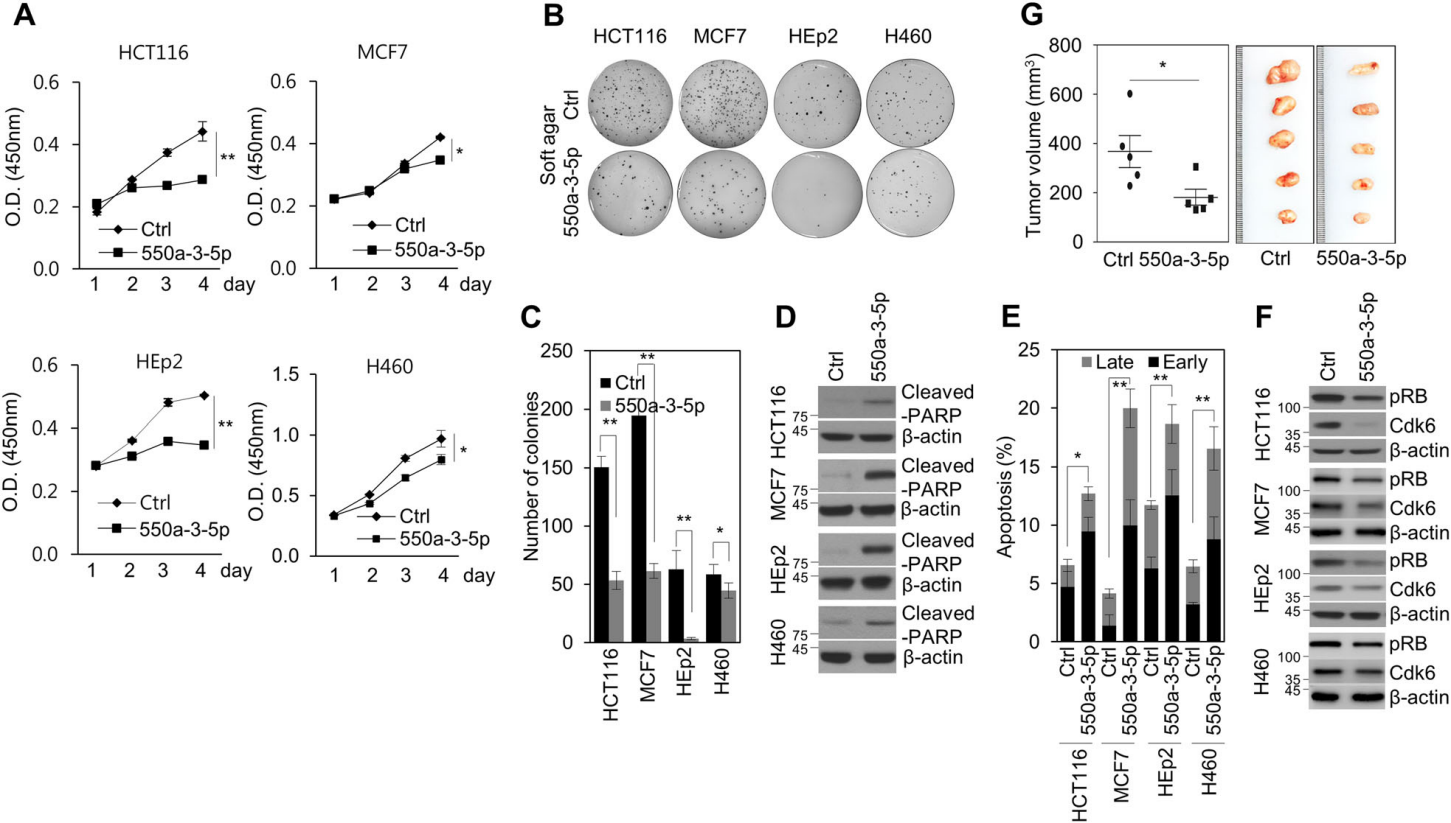
miR-550a-3-5p inhibits cell proliferation and survival in various cancer cells. HCT116, MCF7, HEp-2, and H460 cells were transfected with either control miRNA or miR-550a-3–5p for 48 h. **a** Cell proliferation was determined by WST-8 assay at the indicated day. **b**, **c** Colony formations were determined by soft agar assay. Representative images (**b**) and quantification (**c**) of colony formation by soft agar assay. **d**, **f** The cells were analyzed by immunoblotting with the indicated antibodies. β-actin was used as the loading control. **e** Apoptosis was determined by flow cytometric analysis of FITC Annexin-V. **g** Female BALB/c nude mice were subcutaneously injected with 1 × 10^6^ HCT116 cells. miR-550a-3–5p or control miRNA were intratumorally injected after 13 days. The tumor volume and weight were measured after 29 days. Images of the tumors are shown. The data represent typical results and are presented as the mean ± standard deviation of three independent experiments; *P* < 0.01 (**) and *P* < 0.05 (*)

**Fig. 2:**
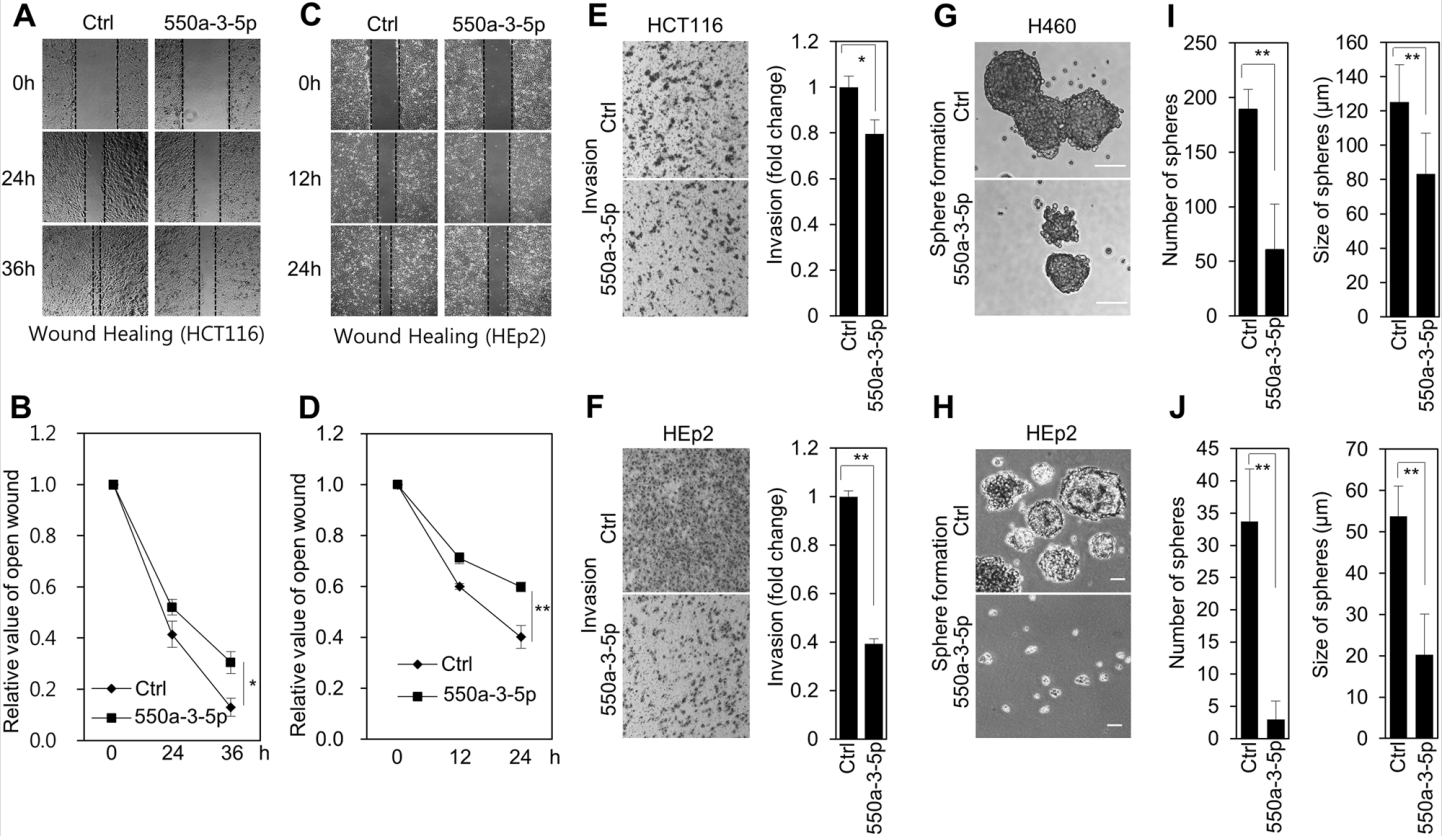
miR-550a-3-5p inhibits migration, invasion, sphere formation in various cancer cells. **a**–**f** HCT116 and HEp-2 cells were transfected with either control miRNA or miR-550a-3-5p for 48 h. **a**–**d** The wound healing was determined at the indicated time points. The wound gaps were photographed and measured. **e**, **f** The transwell invasion assay was performed. Representative images (left panel) and relative quantification (right panel) of the invasive cells on the membrane. **g**–**j** H460 and HEp-2 cells were transfected with either control miRNA or miR-550a-3–5p for 48 h. Representative images (**g** and **h**) and quantification (**i** and **j**) of tumor sphere formation of H460 and HEp-2 cells. The data represent typical results and are presented as the mean ± standard deviation of three independent experiments; *P* < 0.01 (**) and *P* < 0.05 (*)

As miR-550a-3-5p, a novel miRNA, was screened as one of the possible growth-inhibitory miRNAs in HCT116 colon cancer cells^14^, the role of miR-550a-3-5p was examined in multiple human cancer cell lines to determine any possible tumor-suppressive activity. We found that miR-550a-3-5p overexpression significantly reduced cell proliferation (Fig. 1a and Supplementary Fig. S1) and soft agar colony-formation of various cancer cells, including HCT116 colon cancer cells, MCF7 breast cancer cells, HEp-2 laryngeal cancer cell, and H460 lung cancer cells (Fig. 1b, c). In addition, miR-550a-3-5p overexpression increased levels of cleaved-PARP and annexin V, markers of apoptosis (Fig. 1d, e), and decreased the levels of phospho-Rb and CDK6, which was indicative of G1 cell cycle arrest (Fig. 1f). Moreover, we found that miR-550a-3-5p overexpression reduced tumor growth in the HCT116 xenograft model (Fig. 1g), which suggested that miR-550a-3-5p inhibited cancer cell proliferation. Next, we further examined the role of miR-550a-3-5p in tumor migration, invasion, and tumor sphere formation. Interestingly, we found that miR-550a-3-5p overexpression also reduced migration (Fig. 2a–d) and invasion (Fig. 2e, f) in HCT116 and HEp-2 cells. In addition, miR-550a-3-5p overexpression inhibited tumor sphere formation in H460 (Fig. 2g, i) and HEp-2 cells (Fig. 2h, j). For the tumor sphere forming assay, H460 and HEp-2 cells were used because of their high activity of tumor sphere formation. Therefore, our data indicated that the novel miR-550a-3-5p has an antitumor activity in various cancer cells.

### YAP is a direct target of miR-550a-3-5p

**Fig. 3:**
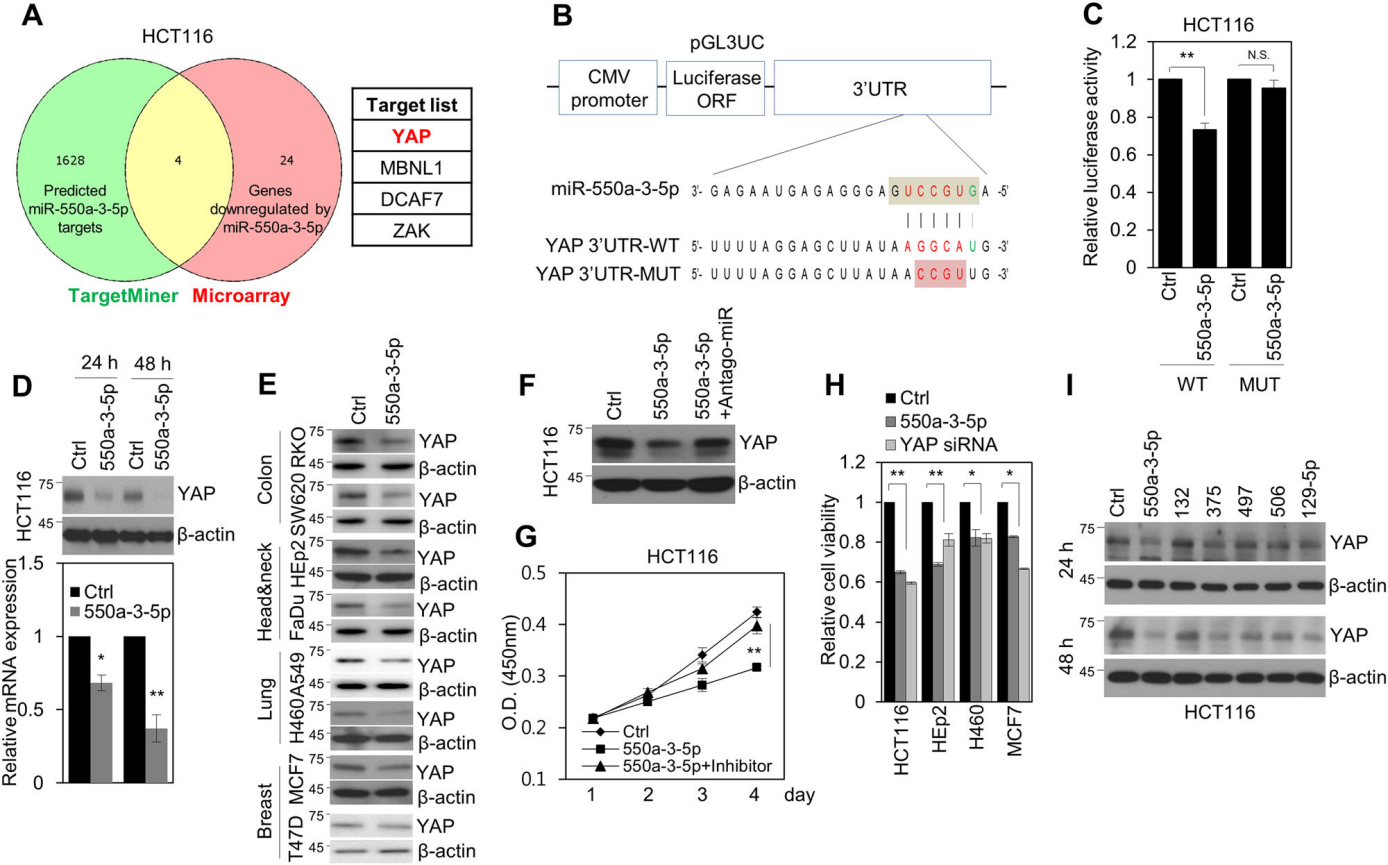
miR-550a-3-5p directly targets oncogenic YAP. **a** Scheme for the identification of candidate genes combining the target prediction algorithm, TargetMiner, and microarray analysis (left panel). The overlapping target genes are listed in the right panel. **b** The putative binding sequences for miR-550a-3-5p within human YAP 3′ UTR wild type (WT) and the mutated (MUT) sequence. Seed sequences are highlighted in gray. **c** HCT116 cells were co-transfected with the indicated reporters and control miRNA or miR-550a-3-5p. Luciferase assay was determined after 24 h transfection. **d** HCT116 cells were transfected with control miRNA or miR-550a-3-5p for 24 or 48 h. YAP expression was determined by western blot analysis (upper panel) and qRT-PCR analysis (lower panel). **e** Various cancer cell lines, including RKO, SW620, HEp-2, FaDu, A549, H460, MCF7, and T47D cells, were transfected with control miRNA or miR-550a-3-5p for 48 h and then analyzed by immunoblotting with YAP antibody. **f**, **g** HCT116 cells were transfected with control miRNA, miR-550a-3-5p, or miR-550a-3-5p with antago-miR-550a-3-5p and then analyzed by immunoblotting with YAP antibody (**f**). Cell proliferation was determined by WST-8 assay on the indicated day (**g**). **h** HCT116, HEp-2, H460, and MCF7 cells were transfected with control miRNA, miR-550a-3-5p, or YAP siRNA for 48 h and cell proliferation was determined by the WST-8 assay. **i** HCT116 cells were transfected with the indicated miRNAs for 24 h (upper panel) or 48 h (lower panel) and the cells were analyzed by immunoblotting with YAP antibody. GAPDH (**d**) or β-actin (**d**, **e**, **f**, and **i**) was used as a loading control. The data represent typical results and are presented as the mean ± standard deviation of three independent experiments; *P* < 0.01 (**) and *P* < 0.05 (*). N.S. not significant

To identify the targets of miR-550a-3-5p, we conducted an in silico search by using TargetMiner and performed a microarray analysis of miR-550a-3-5p-treated HCT116 cells. Among the putative targets and genes downregulated by miR-550a-3-5p, four candidate genes (*YAP*, *MBN1*, *DCAF7*, and *ZAK*) were commonly regulated (Fig. 3a). Of these genes, YAP was selected as a result of its oncogenic properties in multiple cancers^2^. We found base pairing between mature miR-550a-3-5p and the 3′ UTR region of the YAP mRNA ‘seed sequence’ (Fig. 3b). To confirm whether YAP was a direct target of miR-550a-3-5p, wild-type (YAP 3′ UTR-WT) and mutant (YAP 3′ UTR-MUT) 3′ UTR regions of the YAP gene were separately cloned into the pGL3 vector downstream of the luciferase-coding region (Fig. 3b). The luciferase reporter assay indicated that miR-550a-3-5p reduced the luciferase activity of the WT 3′ UTR reporter in HCT116 cells, but had no effect on the MUT 3′ UTR reporter (Fig. 3c); this suggested that YAP was directly regulated by miR-550a-3-5p.

Next, we further examined whether miR-550a-3-5p downregulated endogenous YAP expression and found that miR-550a-3-5p overexpression was able to downregulate YAP mRNA and protein levels after a relatively short time (24 h) in HCT116 cells (Fig. 3d) and confirmed its YAP targeting in various types of cancer cells, including colon cancer (RKO and SW620 cells), head and neck cancer (Fadu and HEp-2 cells), lung cancer (H460 and A549 cells), and breast cancer (MCF7 and T47D cells) (Fig. 3e). In addition, antago-miR-550a-3-5p treatment rescued the miR-550a-3-5p-induced cellular phenotype, such as decreased YAP expression and reduced cell proliferation (Fig. 3f, g). Furthermore, the cytotoxic effect of miR-550a-3-5p was similar to the effect of YAP siRNA in various cancer cells (Fig. 3h). The efficacy of miR-550a-3-5p was also tested by using other miRNAs that have been reported to target YAP in various cancer cells^15–19^. Notably, miR-550a-3-5p was able to efficiently downregulate YAP after a relatively short time (24 h) in comparison with other miRNAs in HCT116 and RKO cells (Fig. 3i), which indicated the supremacy of miR-550a-3-5p-mediated YAP targeting. Collectively, our data suggested that miR-550a-3-5p exerted tumor-suppressive activity through YAP inhibition in multiple cancer cell lines.

### miR-550a-3-5p suppresses oncogenic YAP pathway

**Fig. 4:**
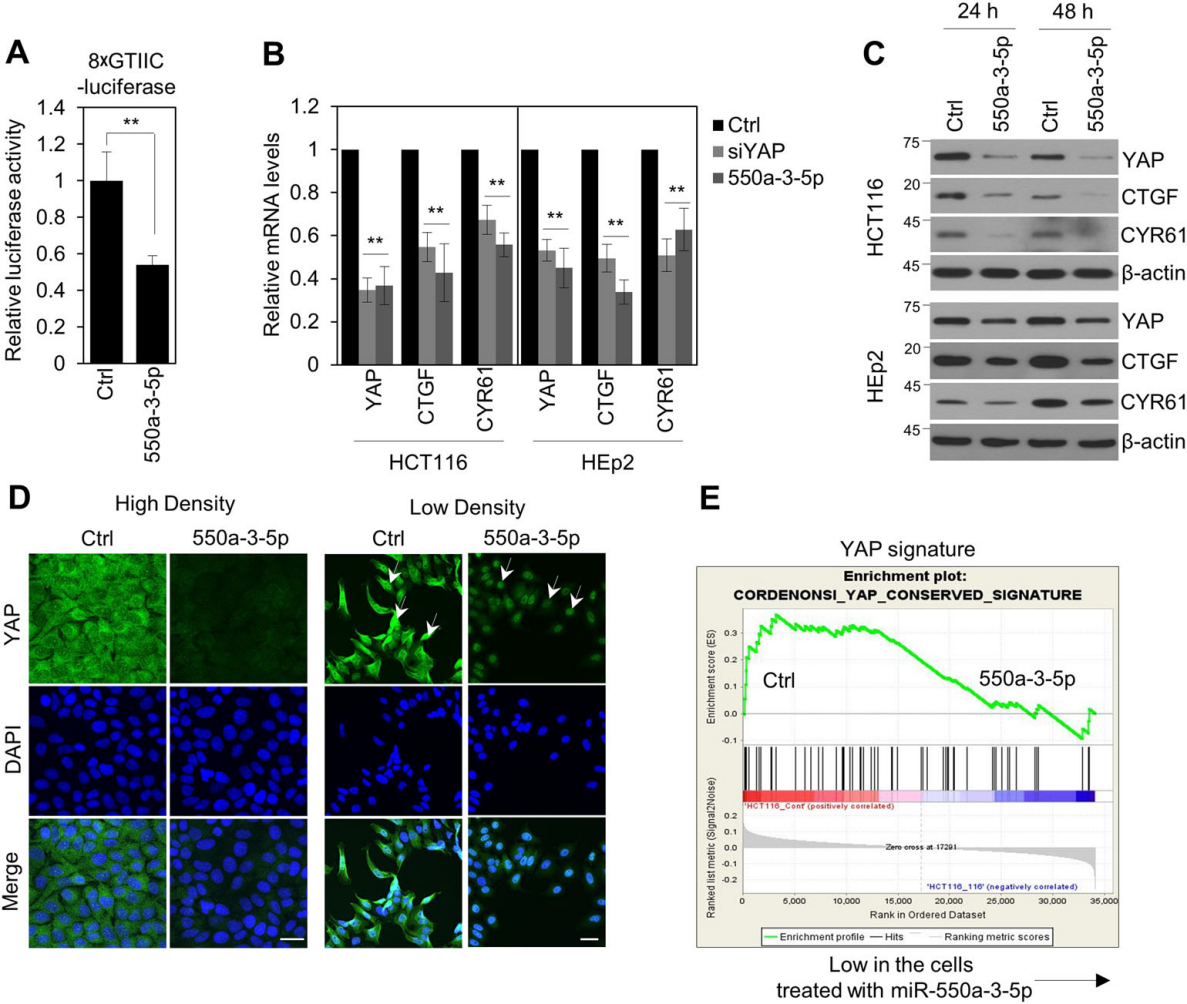
miR-550a-3-5p suppresses oncogenic YAP pathway. **a** HCT116 cells were co-transfected with 8xGTIIC-luciferase vector and control miRNA or miR-550a-3-5p. Luciferase assay was determined after 48 h transfection. **b**, **c** HCT116 and HEp-2 cells were transfected with control miRNA, YAP siRNA, or miR-550a-3-5p for 24 h (**c**) or 48 h (**b** and **c**). The mRNA (**b**) and protein (**c**) expression of YAP, CTGF and CYR61 were measured by qRT-PCR or immunoblotting, respectively, with the indicated antibodies. GAPDH (**b**) or β-actin (**c**) was used as a loading control. **d** 1 × 10^4^ (high density) or 1.25 × 10^3^ (low density) HCT116 cells were seeded and transfected with control miRNA or miR-550a-3-5p for 48 h. The cells were stained with anti-YAP antibody (green) and DAPI (DNA; blue). The arrows indicate the nuclear localized YAP. Scale bars, 30 μm. **e** GSEA enrichment plots show the downregulation of YAP signature genes in the miR-550a-3-5p-transfected cells. The data represent typical results and are presented as the mean ± standard deviation of three independent experiments; *P* < 0.01 (**)

To further understand the role of miR-550a-3-5p in the regulation of the oncogenic YAP pathway, we next examined whether miR-550a-3-5p modulated the YAP pathway. First, we used a YAP/TAZ-responsive TEAD reporter (8xGTIIC-luciferase reporter) that contained eight TEAD-binding elements^20^. The reporter activity showed that miR-550a-3-5p overexpression suppressed YAP/TAZ transcriptional activity in HCT119 cells (Fig. 4a). In addition, miR-550a-3-5p overexpression decreased the mRNA and protein levels of CTGF and CYR61, direct target genes of YAP^2^, in HCT116 and HEp2 cells (Fig. 4b, c), which indicated that miR-550a-3-5p inhibited the YAP pathway. Next, to check whether miR-550a-3-5p modulated YAP/TAZ nuclear translocation activity in addition to the regulation of YAP expression, immunofluorescence staining for YAP was performed in high density culture and low density culture of HEp-2 cells treated with control miRNA or miR-550a-3-5p. As expected, YAP was predominantly localized in the cytoplasm in high-density cultured cells (Fig. 4d, left control panel) and in the nucleus in the low-density cultured cells (Fig. 4d, right control panel). miR-550a-3-5p overexpression completely suppressed cytoplasmic YAP, but not nuclear YAP (Fig. 4d, miR-550a-3-5p-treated panel), which indicated that miR-550a-3-5p suppressed cellular YAP. In addition, we also performed GSEA by using the microarray data to compare the control miRNA- versus miR-550a-3-5p-treated HCT116 cells. The GSEA using MSigDB (C6 oncogenic signature) revealed a decrease of the YAP signature in miR-550a-3-5p-treated HCT116 cells (nominal *P*-value < 0.05) (Fig. 4e). Therefore, our data indicated that miR-550a-3-5p inhibited the oncogenic YAP pathway through the suppression of cellular YAP.

### Inverse correlation between YAP and miR-550a-3-5p expression occurs in colon cancer tissues and in a density-dependent manner

**Fig. 5:**
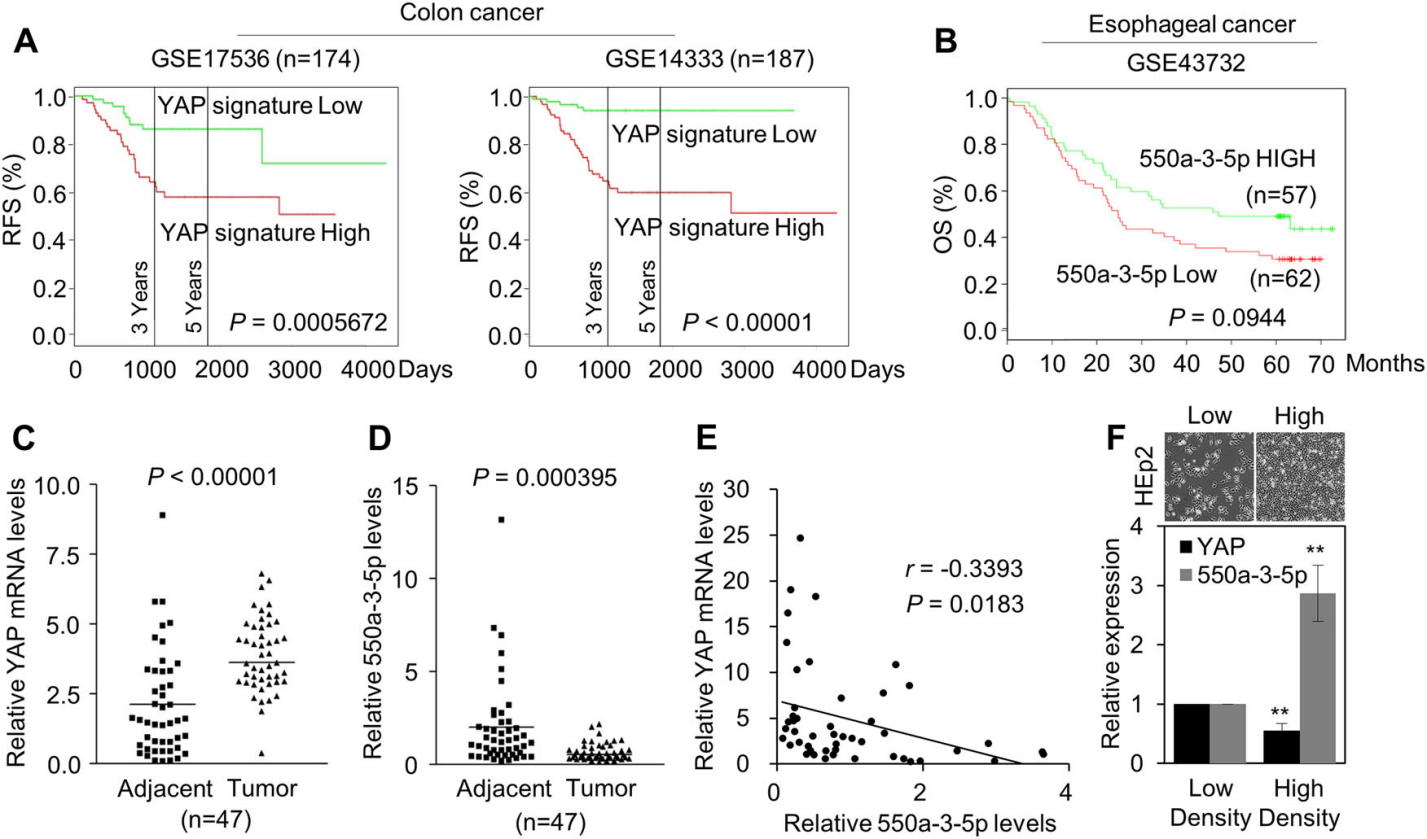
Inverse correlation between YAP and miR-550a-3-5p in colon cancer tissues and in a density-dependent manner. **a** Kaplan-Meier survival curves for recurrence-free survival of colon cancer patients by high or low YAP signature. YAP signature (*YAP*, *CTGF*, and *CYR61*) levels in the cohort of colon cancer patients were obtained from GEO database (GSE17536 and GSE14333) and analyzed using PROGgene. **b** Kaplan-Meier survival curves for overall survival of the GSE43732 dataset by high or low miR-550a-3-5p. **c**, **d** Expression levels of YAP mRNA (**c**) or miR-550a-3-5p (**d**) in colon cancers and their adjacent normal tissue counterparts. **e** Inverse correlation between YAP mRNA and miR-550a-3-5p in colon cancer tissues. **f** 8 × 10^5^ HEp-2 cells were cultured in a 100-mm dish (low density) or 35-mm dish (high density) for 24 h. YAP or miR-550a-3-5p expression was analyzed by qRT-PCR (lower panel). GAPDH or U6 was used as the loading control. Representative images of low or high density cultured cells (upper panel). The data represent typical results and are presented as the mean ± standard deviation of three independent experiments; *P* < 0.01 (**)

To address whether the YAP pathway was associated with miR-550a-3-5p in tumor patients, we first evaluated the clinical significance of the YAP pathway by using a colon cancer public database and tumor specimens. First, to check whether the YAP pathway was associated with poor colon cancer survival, the PROGene database, which contains tools to enable multiple gene-based prognostic assessment^21^, was used to determine the correlation between the survival outcome and the YAP signature (*YAP1*, *CTGF*, and *CYR61*) in colon cancer. Notably, we found that the high YAP signature was strongly correlated with poor survival in the Moffitt dataset^22^ and the Ludwig dataset (Fig. 5a)^23^, which indicated that the activation of the YAP pathway is associated with the poor prognosis of colon cancer. With regard to the prognostic value of miR-550a-3-5p, we were able to determine that the high levels of miR-550a-3-5p were associated with good prognosis in esophageal cancer^24^ (Fig. 5b) and supported the tumor suppressive role of miR-550a-3-5p in multiple cancers. Next, we further examined the correlation between YAP and miR-550a-3-5p expression in 47 colon cancer specimens and their paired adjacent noncancerous specimens. YAP mRNA levels were upregulated in colon cancer tissues in comparison with their respective adjacent noncancerous specimens, whereas miR-550a-3-5p levels were downregulated (Fig. 5c and d). Similar results were also observed between normal colon cells and colon cancer cell lines (Supplementary Fig. S2a). In addition, YAP mRNA levels were inversely correlated with miR-550a-3-5p levels (Fig. 5e). Furthermore, CTGF mRNA levels, which were positively correlated with YAP mRNA levels (Supplementary Fig. S2b), was also inversely correlated with miR-550a-3-5p levels in colon cancer tissues (Supplementary Fig. S2c).

As it has been reported that many tumor suppressive miRNAs are epigenetically inactivated in tumor tissues and that YAP regulated miRNA biogenesis in a cell density-dependent manner^7,25^, we further examined whether the decreased miR-550a-3-5p in cancer cells was controlled by epigenetic modification or density-dependent regulation. We found that treatment with 5-aza-2-deoxycytidine, a demethylating agent, did not modulate the expression of miR-550a-3-5p in HCT116 and HEp-2 cells (Supplementary Fig. S3). Instead, miR-550a-3-5p was increased in the high-density HEp-2 culture, whereas YAP was decreased in the same conditions (Fig. 5f), which suggested that the decrease of miR-550a-3-5p in cancer cells could be regulated by a density-dependent manner. Therefore, our data indicated that the inverse correlation between YAP and miR-550a-3-5p expression in colon cancer tissues and that this inverse correlation was regulated in a density-dependent manner, which provided evidence of the clinical relevance for the role of miR-550a-3-5p in YAP regulation in human cancers.

### miR-550a-3-5p sensitizes BRAF inhibitor resistance through YAP inhibition with reduced AKT activity in BRAF-mutant colon cancer and melanoma cells

**Fig. 6:**
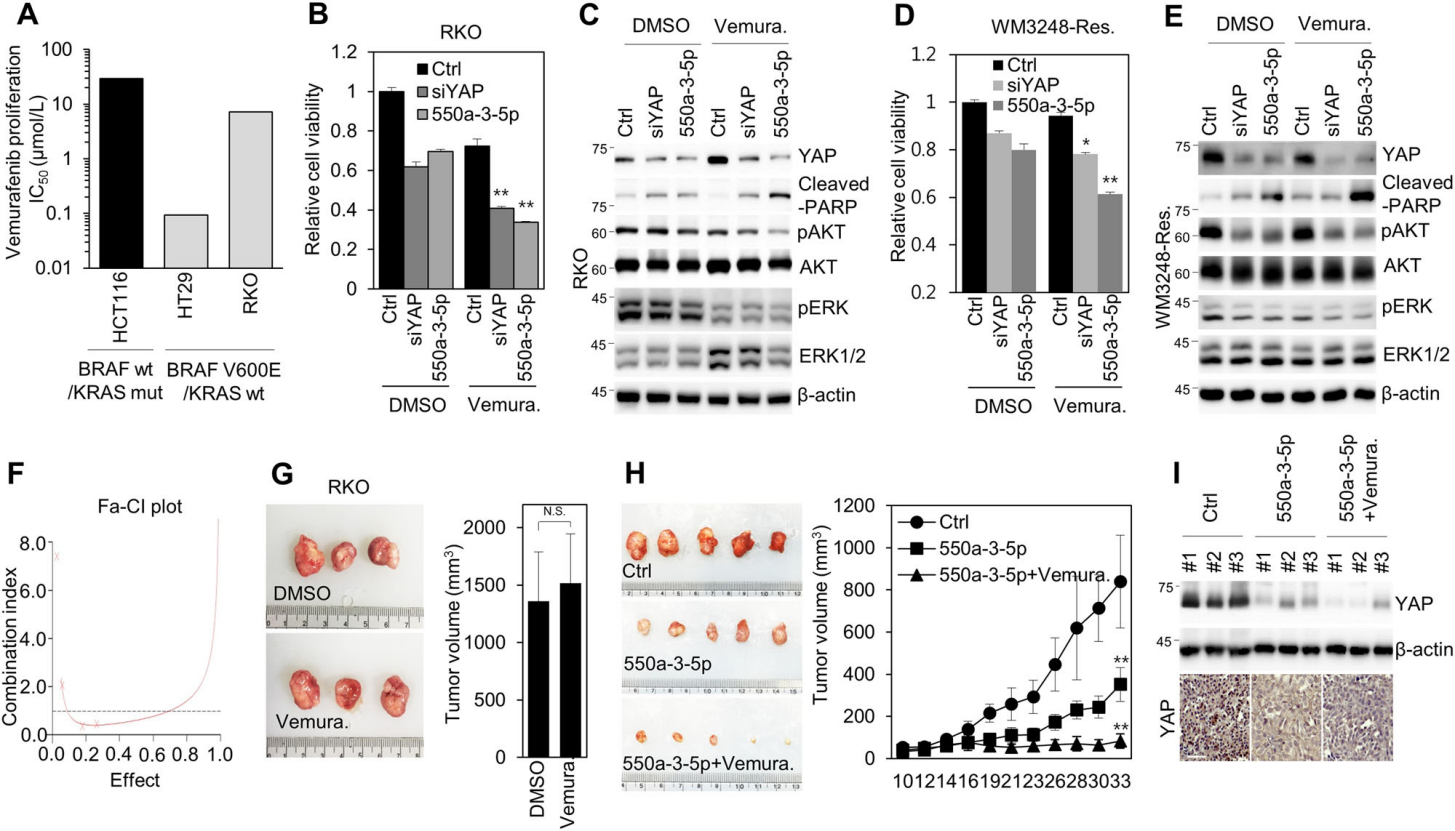
miR-550a-3-5p sensitizes BRAF-inhibitor resistant colon cancer and melanoma cells. **a** HCT116, HT29, and RKO cells were treated with the various concentration of vemurafenib to determine the IC_50_. The cell viabilities were determined by WST-8 assay. **b**, **c** RKO cells were transfected with either control miRNA, YAP siRNA, or miR-550a-3-5p for 48 h and then treated with 2 μM vemurafenib or DMSO for a further 24 h. The cell viabilities were determined by WST-8 assay (**b**). The cells were analyzed by immunoblotting with the indicated antibodies (**c**). **d**, **e** Vemurafenib-resistant W3248 cells were transfected with control miRNA, YAP siRNA,or miR-550a-3-5p for 48 h. Cell viabilities were determined by WST-8 assay (**d**). The cells were analyzed by immunoblotting with the indicated antibodies (**e**). β-Actin was used as the loading control (**c** and **e**). **f** The combination index was measured by Calcusyn. **g**, **h** Female BALB/c nude mice were subcutaneously injected with 1 × 10^6^ RKO cells. **g** The mice were treated with DMSO or vemurafenib (75 mg/kg daily). **h** The mice were treated with control miRNA and vehicle, miR-550a-3-5p and vehicle, or miR-550a-3-5p and vemurafenib (75 mg/kg daily). Images of the tumors are shown (**g** and **h**, left panels). The tumor volume and weight were calculated over time as indicated (**g** and **h**, right panels). **i** YAP expression in the tumor tissues were analyzed by immunoblotting (upper panel) and immunohistochemistry (lower panel) with the YAP antibody; β-actin was used as the loading control. Scale bars, 100 μm. The data represent typical results and are presented as the mean ± standard deviation of three independent experiments; *P* < 0.01 (**) and *P* < 0.05 (*). N.S. not significant

As it has been reported that YAP activation is a critical factor for BRAF inhibitor resistance in BRAF-mutant cancer cells^5,6^ and that the inhibition of the PI3K/AKT pathway is a key mechanism to overcome BRAF inhibitor resistance in BRAF-mutant colon cancer cells^26,27^, we subsequently examined the possibility that miR-550a-3-5p treatment could overcome BRAF inhibitor resistance through the inhibition of AKT activity in BRAF inhibitor-resistant colon cancer and melanoma cells. To test this possibility, RKO cells harboring BRAF^V600E^/KRAS^WT^ were utilized because RKO cells have high intrinsic resistance to vemurafenib (BRAF^V600E^ selective inhibitor) compared with HT29 cells, which also harbor BRAF^V600E^/KRAS^WT^ (Fig. 6a)^27^. HCT116 cells harboring BRAF^WT^/KRAS^mut^ that are insensitive to vemurafenib were used as a negative control (Fig. 6a). Consistent with previous report^27^, we also confirmed that RKO cells exhibited increased levels of phosphorylated AKT and ERK in comparison with HT29 cells, but not EGFR, the expression of which is known to be associated with vemurafenib resistance (Supplementary Fig. S4a)^28^. In addition, we observed that YAP was more strongly active in RKO cells in comparison with HCT116 and HT29 cells, as evidenced by the decreased inhibitory phosphorylation of YAP, an indicative of YAP activation, and increased expression of Bcl-xl, a YAP target gene (Supplementary Fig. S4b). Notably, we found that miR-550a-3-5p treatment sensitized the vemurafenib resistance of RKO cells through the reduced phosphorylation of AKT, but did not modulate phosphorylated ERK in RKO cells (Fig. 6b, c), which was also observed in YAP siRNA-treated cells.

As BRAF mutation is commonly observed in patients with melanoma and its resistance mechanism is associated with YAP activation^3,5^, vemurafenib-resistant WM3248 melanoma cells, which are cells with acquired resistance^6^, were utilized to further examine the role of miR-550a-3-5p in BRAF inhibitor resistance. Consistent with a previous report^6^, we also observed that the resistant WM3248 cells showed a higher level of EGFR and maintained ERK/AKT activity in response to vemurafenib treatment than the parental control cells (Supplementary Fig. S4c). Importantly, we found that miR-550a-3-5p treatment increased vemurafenib sensitivity through the reduced phosphorylation of AKT, but marginally modulated phosphorylated ERK in the resistant WM3248 cells (Fig. 6d, e), which was consistent with the results in RKO cells. The analysis of the combination index also indicated that the combination treatment of miR-550a-3-5p and vemurafenib exerted synergistic effects in resistant WM3248 cells (Fig. 6f). Moreover, we further validated the inhibitory effect of miR-550a-3-5p on BRAF inhibitor resistance by using an RKO xenograft model. Similar to the in vitro results, vemurafenib treatment was insensitive to tumor growth in RKO xenograft model (Fig. 6g), whereas the combination treatment of miR-550a-3-5p and vemurafenib sensitized the vemurafenib resistance of the RKO xenograft model through YAP inhibition (Fig. 6h, i). Collectively, our data suggested that miR-550a-3-5p treatment sensitized BRAF inhibitor resistance through YAP inhibition with reduced AKT activity in BRAF-mutant colon cancer and melanoma cells, which provided evidence for the potential therapeutic role of miR-550a-3-5p in BRAF inhibitor-resistant colon cancers and melanomas.

## Discussion

Our data showed that the novel miR-550a-3-5p suppressed cell proliferation, metastasis, and tumor sphere formation through the direct inhibition of oncogenic YAP in various cancer cell types. In addition, we demonstrated the clinical relevance of miR-550a-3-5p-mediated YAP regulation in multiple cancers, including an inverse correlation between miR-550a-3-5p and YAP signature in colon cancers and its prognostic value in esophageal cancer. Importantly, we found that miR-550a-3-5p treatment sensitized vemurafenib resistance through YAP inhibition with reduced AKT activity in vemurafenib-resistant colon cancer and melanoma cells. Therefore, our data provide the first evidence for the potential role of the novel miR-550a-3-5p in YAP-mediated tumorigenesis and BRAF inhibitor resistance.

It is well established that miRNA plays the role of an oncogene or a tumor suppressor, depending on its target mRNAs, in various different cancers^7^. Our results revealed that the novel miR-550a-3-5p exerted its tumor suppressive role through the targeting of YAP in various cancer cells. We showed that miR-550a-3-5p overexpression inhibited various oncogenic properties, including cell proliferation, anti-apoptosis, migration, invasion, and cancer stemness, which was consistent with the effects of YAP inhibition in various cancer cells (Figs. 3h, 4b)^1,2,29^. Interestingly, it was recently reported that miR-550a-3p, the 3′ antisense strand of miR-550a-3, exerts tumor suppressive activity through the targeting of ERK1/2 in breast cancer^30^. Similar to miR-550a-3-5p, miR-550a-3p expression was reduced in breast cancer tissues, whereas the ectopic overexpression of miR-550a-3p inhibited the proliferation of breast cancer cells^30^. Thus, it was likely that miR-550a-3 might be a functional tumor suppressor through the inhibition of key oncogenes such as YAP and ERK in multiple cancers.

Several pieces of evidences from our data, including luciferase activity assay, western blot analysis, YAP reporter assay, target gene expression analysis, and GSEA, clearly indicated that YAP was a bona fide target of miR-550a-3-5p. Although several miRNAs have been reported to regulate YAP expression in various cancer cells, these YAP-regulatory miRNAs examined in small numbers of cancer types, such as liver cancer, NSCLC, or ovarian cancer^15–19^. However, we showed that miR-550a-3-5p downregulated YAP in various types of cancer cells, including colon, head and neck, lung, and breast cancer (Fig. 3e). In addition, miR-550a-3-5p was able to inhibit YAP in a relatively short time (24 h) compared with other miRNAs (Fig. 3i), which supported the miR-550a-3-5p targeting of YAP and exerts its tumor-suppressive role in multiple cancers.

We also showed that reduced miR-550a-3-5p levels were inversely correlated with YAP in colon cancer tissues and this inverse correlation was regulated by density-dependent culture. As several studies have shown that miRNA expression was globally suppressed in tumor cells^7^ and that YAP overexpression suppressed miRNA biogenesis in a cell density-dependent manner^25^, it may be possible that YAP activation in the early stage of tumorigenesis suppressed YAP-inhibitory miRNAs and led to an increase in YAP expression as a positive feedback mechanism. Indeed, our results showed that miR-550a-3-5p expression was regulated by a density-dependent cell culture condition (Fig. 5f). Similarly, recent reports indicated that a density-sensitive miRNA downregulated YAP expression in colon cancer^31^. Furthermore, we showed that decreased miR-550a-3-5p was not mediated by hypermethylation in HCT116 and HEp-2 cells. Therefore, it was possible that the reduction of miR-550a-3-5p might be associated with YAP overexpression as a positive feedback mechanism in multiple cancers.

Constitutive activation mutations in BRAF promote malignant tumorigenesis through the activation of downstream signaling via MEK and ERK^32^. Oncogenic BRAF activation is implicated in approximately 50% of melanomas and between 8 and 10% of colon cancers^27,32^. Although selective BRAF mutant inhibitors have been developed, such as vemurafenib and dabrafenib, acquired or intrinsic drug resistance is a major problem for the treatment of BRAF-mutant cancer patients. In this study, we utilized RKO colon cancer cells as the intrinsic resistant cells and vemurafenib-resistant WM3248 melanoma cells as the acquired resistant cells to vemurafenib. Notably, we demonstrated that miR-550a-3-5p treatment sensitized BRAF inhibitor-resistant colon and melanoma cancer cells through YAP inhibition with reduced AKT activity, which suggested that miR-550a-3-5p treatment could be useful for the treatment of acquired and intrinsic vemurafenib resistance. Consistent with our observation, it is known that AKT activation is an important bypass mechanism for acquired and intrinsic vemurafenib resistance in melanomas and colon cancers^26,27,32^. In addition, the bioinformatic analysis for miR-550a-3-5p pathway by using the DIANA tool^33^ (Supplementary Fig. S5) suggested that miR-550a-3-5p could largely modulate the PI3K/AKT pathway. Furthermore, the recent study suggested that YAP mediated crosstalk between the Hippo and PI3K-TOR pathways^34^. Therefore, it is possible that miR-550a-3-5p exerts direct inhibition of oncogenic YAP and subsequently perturbs the PI3K/AKT pathway in BRAF mutant cancer cells.

In conclusion, our results suggested that the novel miR-550a-3-5p acts as a tumor suppressor and reverses BRAF inhibitor resistance through the direct regulation of YAP in multiple cancer cells. Our work provides new evidence of the miRNA-mediated YAP regulation in tumor progression and resistance.

## Materials and methods

### Cell culture and treatment

All cancer cell lines were purchased from American Type Culture Collection (ATCC; Manassas, VA) between February 2013 and July 2014. As indicated in the provided information, all cell lines were authenticated by their karyotypes, images and detailed gene expression. The cells were preserved and passaged in accordance with ATCC protocols for a maximum of 2 months and tested weekly for mycoplasma infection by PCR method. Cells were cultured in DMEM (Gibco-BRL, NY) supplemented with 10% FBS (Corning, NY) and 1% penicillin/streptomycin at 37 °C in a humidified 5% CO_2_ incubator. Dr. Joon Kim (Korea Advanced Institute of Science and Technology, Daejeon, Korea) provided the parental WM3248 melanoma cells and vemurafenib-resistant WM3248 melanoma cells^6^. The parental WM3248 cells and vemurafenib-resistant WM3248 cells were grown in MEM (Gibco-BRL) supplemented with 10% FBS; vemurafenib-resistant WM3248 cells were maintained in the presence of 2 μM vemurafenib (Adooq Bioscience, CA). Before the experiments, cell number and viability were measured using a Luna II automated cell counter (Logos Biosystems, Anyang, Korea).

#### Cell proliferation and viability assay

The cells were treated with the indicated conditions in 96-well plates. Cell proliferation or viability was determined by using the WST-8 assay (Cyto X^TM^ cell viability assay kit; LPS solution, Daejeon, Korea) or BrdU cell proliferation assay (Cell signaling technology, Inc., Danvers, MA) in accordance with the manufacturer’s protocol. The absorbance was measured at 450 nm by using a Versamax microplate reader (Molecular Devices, CA).

### Apoptosis assay

Cells were treated with the indicated conditions in 6-well plates. After 48 h, the cells were collected and detected using FITC Annexin V Apoptosis Detection Kit (BD biosciences, CA) in accordance with the manufacture’s protocol. The samples were analyzed by a BD Accuri C6 flow cytometer using the C6 Software.

### Soft agar assay

Cells were transfected with 20 nM control miRNA or 20 nM miR-550a-3-5p. After 48 h, cells were trypsinized, gently mixed with 0.45% agar medium mixture (Difco Noble Agar, BD Biosciences) and re-seeded on 6-well plates covered with a layer of 0.9% agar in DMEM medium. After 2 weeks, the colonies were stained with 0.2% crystal violet (Sigma-Aldrich, St Louis, MO) and quantified by colony counting.

### Wound healing and invasion assay

For the wound healing assay, the cells treated with indicated conditions were seeded and grown to confluence in 6-well plates. The cell monolayers were scratched with a 10 μL pipette tip. Wound fields were photographed at 0, 12, 24, and 36 h after wounding at three different sites. The invasion assay was analyzed by using the Transwell chamber assay (Costar, MA). The cells (5 × 10^4^ cells) treated with indicated conditions were seeded on the upper insert coated with 2% Matrigel (Corning) in 24-well plates and the lower chamber was filled with 600 μL DMEM supplemented with 10% FBS. After 12 h (HEp-2 cells) or 24 h (HCT116 cells) of incubation, the number of invaded cells were quantified in five random fields.

### Sphere formation assay

Cells were transfected with 20 nM control miRNA or 20 nM miR-550a-3-5p. After 48 h, 2500 cells were plated on ultra-low attachment plates (Corning) in serum-free DMEM-F12 medium (Gibco-BRL) supplemented with B-27 (1:50; Invitrogen, CA), 20 ng/mL FGF (R&D Systems, MN), and 20 ng/mL EGF (R&D Systems). After 10–14 days, the sphere was fixed with methanol. The average sphere number and size were analyzed by ImageJ software. Spheres with a diameter below 50 mm were excluded from the analysis.

### RNA interference

The following sequences were used for RNA interference: YAP, 5′-CAGAAGAUCAAAGCUACUU-3′; Hsa-miR-550a-3-5p, 5′-AGUGCCUGAGGGAGUAAGAG-3′; and miR-550a-3-5p inhibitor targeting miR-550a-3-5p was purchased from IDT (Integrated DNA Technologies, IA). Nonsilencing siRNA (Bioneer, Daejeon, Korea) or control miRNA (Genolution, Seoul, Korea) was used as a negative control. The transfection of siRNAs or miRNAs (20 nM YAP siRNA and 20 nM hsa-miR-550a-3-5p) was conducted by using G-Fectin (Genolution) in accordance with the manufacturer’s protocol.

### Antibodies and western blot analysis

The following antibodies were used: mouse monoclonal antibodies against CYR61 (A-10), phospho-ERK1/2 (E-4), and β-actin (C4; Santa Cruz Biotechnology, Inc., Santa Cruz, CA); CTGF (Abgent, CA); rabbit monoclonal antibodies against Bcl-xl (54H6), cleaved-PARP (Asp214, #5625), and phospho-AKT (EP2109Y) (Cell Signaling Technology, Inc.); rabbit polyclonal antibodies against CDK6 (C-21), EGFR, phospho-RB (Ser249/Thr252), and YAP (G-6; Santa Cruz Biotechnology, Inc); AKT, and ERK1/2 (Cell Signaling Technology, Inc.).

Western blotting was performed, as previously described^35^. In brief, proteins were separated by SDS-polyacrylamide gel electrophoresis, transferred to a polyvinylidene fluoride membrane, and incubated with specific antibodies. The HRP-conjugated secondary antibody was detected by using an enhanced chemiluminescence detection system (Amersham Life Science, NJ, USA) and the ban were imaged by using the Amersham Imager 600 system (GE Healthcare) or X-ray films (Agfa-Gaevert, Mortsel, Belgium).

### Immunofluorescence

Immunofluorescence analysis was performed, as previously described^35^. In brief, cells were fixed with 4% paraformaldehyde, permeabilized, and blocked with 0.2% Triton X-100 and 5% fetal calf serum in PBS. The fixed cells were incubated with a primary antibody against YAP (Santa Cruz Biotechnology, Inc.) and a secondary anti-mouse Alexa-488 antibody (Molecular Probes, Eugene, OR). The images were obtained by using a confocal laser-scanning microscope (LSM 710; Carl Zeiss, Inc.).

### Immunohistochemistry

Immunofluorescence analysis was performed in accordance with a previously described protocol^35^. Briefly, immunohistochemical staining was performed by using anti-YAP rabbit polyclonal antibody (G-6; Santa Cruz Biotechnology; 1:100). Immunostaining was detected by the avidin-biotin-peroxidase method in accordance with the manufacturer’s instructions (Invitrogen).

### Microarray and data mining

HCT116 cells were transfected with 20 nM control miRNA or 20 nM miR-550a-3-5p. After 48 h, total RNAs were isolated with TRIzol reagents (Invitrogen). Genome-wide gene expression analysis was performed by using Illumina Human HT-12v4 Expression BeadChip (Illumina, Inc., CA), as previously described^14^. The array data have been deposited into the NCBI GEO database (GSE102571).

TargetMiner (www.isical.ac.in/~btargetmine/targetminer20.htm) or DIANA TOOLS (diana.imis.athena-innovation.gr/DianaTools/index.php) was used to predict the miR-550a-3-5p targets and pathways, respectively. To identify the YAP signature from the microarray data in control miRNA- or miR-550a-3-5p-treated HCT116 cells, Gene Set Enrichment Analysis (GSEA) was performed by using the Broad Institute Platform (www.broadinstitute.org/gsea/index.jsp) in accordance with the GSEA User Guide. The oncogenic signature C6 from the molecular signatures database was used for the analysis. The prognostic value of the YAP signature (*YAP, CTGF*, and *CYR61* mRNAs) in the colon cancers or miR-550a-3-5p in esophageal cancer was assessed by using the PROGgene database (watson.compbio.iupui.edu/chirayu/proggene/database/?url=proggene) or the MIRUMIR database (www.chemoprofiling.org/cgi-bin/GEO/MIRUMIR/web_run_MIRUMIR.V1.pl), respectively.

### DNA construct and luciferase activity assay

The human YAP 3′ UTR containing the putative miR-550a-3-5p binding site was cloned into the XbaI and EcoRV sites of pGL3luc (provided by Dr. V.N. Kim, Seoul National University, Korea). The following primers were used for the amplification of the YAP 3′ UTR: forward, 5′-ACCCGCGG GTTATAGAGCCCTCAGGCAGAC-3′ and reverse, 5′-ACAGATATC TGTAAGTACCTAACATATGAGC-3′. To generate the mutant reporter vector, four nucleotide mutations were introduced into the putative miR-550a-3-5p binding site by using VELOCITY DNA Polymerase (Bioline, USA) in accordance with the QuikChange II Site-Directed Mutagenesis Kit (Agilent Technologies, CA) instruction manual.

8xGTIIC-luciferase plasmid was provided by Dr. Stefano Piccolo (University of Padua; Addgene plasmid #34615). HCT116 cells were transfected with the reporter plasmid (100 ng), pRL-CMV-*Renilla* plasmid (1 ng), and miR-550a-3-5p (20 nM) in 24-well plates by using the TransIT-X2 Delivery System (Mirus, Madison, WI) in accordance with the manufacturer’s protocol. After 24 h, luciferase assays were conducted by using the Dual Luciferase Reporter Assay system (Promega, WI) in accordance with the manufacturer’s instructions. The firefly luciferase activity was normalized to *Renilla* luciferase activity.

### Tissue samples

Forty-seven pairs of colorectal cancer tissues and their matched non-tumor adjacent tissues were acquired from patients undergoing surgery for colorectal cancer at Inje University Paik Hospital (Busan, Korea) and Busan National University Hospital (Busan, Korea). All tissues were immediately frozen and stored in liquid nitrogen until RNA extraction. All patients gave signed, informed consent for their participation in scientific research (IRB number: K-1504-002-044).

### RNA isolation and qRT-PCR

Total RNA was extracted from the cells or tissues with TRIzol reagents (Invitrogen) or Qiazol reagents (Qiagen, Hilden, Germany) in accordance with the manufacturer’s protocol. qRT-PCR was performed to measure mature miRNA expression with the TaqMan Universal Master Mix (Applied Biosystems, CA), or mRNA expression with the KAPA SYBR qPCR Master Mix kit (KAPA Biosystems, MA). The TaqMan primer for miR-550a-3-5p was purchased from Applied Biosystems. GAPDH or control miRNA U6 was used for normalization. The following primers were used for qRT-PCR: CTGF, forward 5′-ACCGACTGGAAGACACGTTTG-3′ and reverse 5′-CCAGGTCAGCTTCGCAAGG-3′; CYR61, forward 5′-GGTCAAAGTTACCGGGCAGT-3′ and reverse 5′-GGAGGCATCGAATCCCAGC-3′; YAP, forward 5′-TAGCCCTGCGTAGCCAGTTA-3′ and reverse 5′-TCATGCTTAGTCCACTGTCTGT-3′.

### Combination index analysis

For the assessment of the synergistic or additive effects of combined miR-550a-3–5p and Vemurafenib treatment, combination index (CI) was used. HCT116 cells were transfected with 20 nM miR-550a-3-5p. After 48 h, vemurafenib was added at four different concentrations (1, 2, 4, and 8 μM). The CI for the miR-550a-3-5p and vemurafenib combination was calculated by median dose-effect analyzes using commercially available software (CalcuSyn, Biosoft, Great Shelford, Cambridge, UK). CI values of 1.0 represent synergistic action of the two drugs in combination.

### Xenograft studies

Four-week-old female BALB/c nude mice were purchased from ORIENT Bio (Seongnam, Korea) and maintained under aseptic conditions for 1 week. HCT116 and RKO cells (1 × 10^6^) were implanted subcutaneously in the right flank of nude mice. The miR-550a-3-5p or control miRNA with in vivo-jetPEI (Polyplus Transfection, NY) complex in a volume of 50 μl (15 μg/site) were injected intratumorally three times per week, between day 14 and 33 after inoculation, in accordance with the manufacturer’s protocol. Vemurafenib was formulated in 5% dimethyl sulfoxide (Sigma-Aldrich), 1% methylcellulose (Sigma-Aldrich); and the animal received vemurafenib (75 mg/kg) daily by oral gavage. Vehicle was administered as the negative control. Tumor volumes were measured three times per week by using a caliper and calculated by (l × w × w)/2. The mice were killed 34 days after the tumor cells were seeded, after which the subcutaneous tumors were excised and weighed. Animal care and treatment followed institutional guidelines.

### Statistical analysis

The correlation between YAP and miR-550a-3-5p was analyzed by using Spearman’s rank correlation test. A two-tailed Student’s *t*-test was performed to assess the statistical differences between groups. *P*-values of <0.05 were considered to be statistically significant. Statistical analyzes were computed by Excel and XLSTAT software.

## Electronic supplementary material

Supplementary Figure 1

Supplementary Figure 2

Supplementary Figure 3

Supplementary Figure 4

Supplementary Figure 5

Supplementary figure legends
