# Supplementary figures and images for: miR-550a-3-5p acts as a tumor suppressor and reverses BRAF inhibitor resistance through the direct targeting of YAP
Source: Cell Death Dis. 2018 May 29;9(6):640. doi: 10.1038/s41419-018-0698-3 (PMC5974323; doi:10.1038/s41419-018-0698-3)

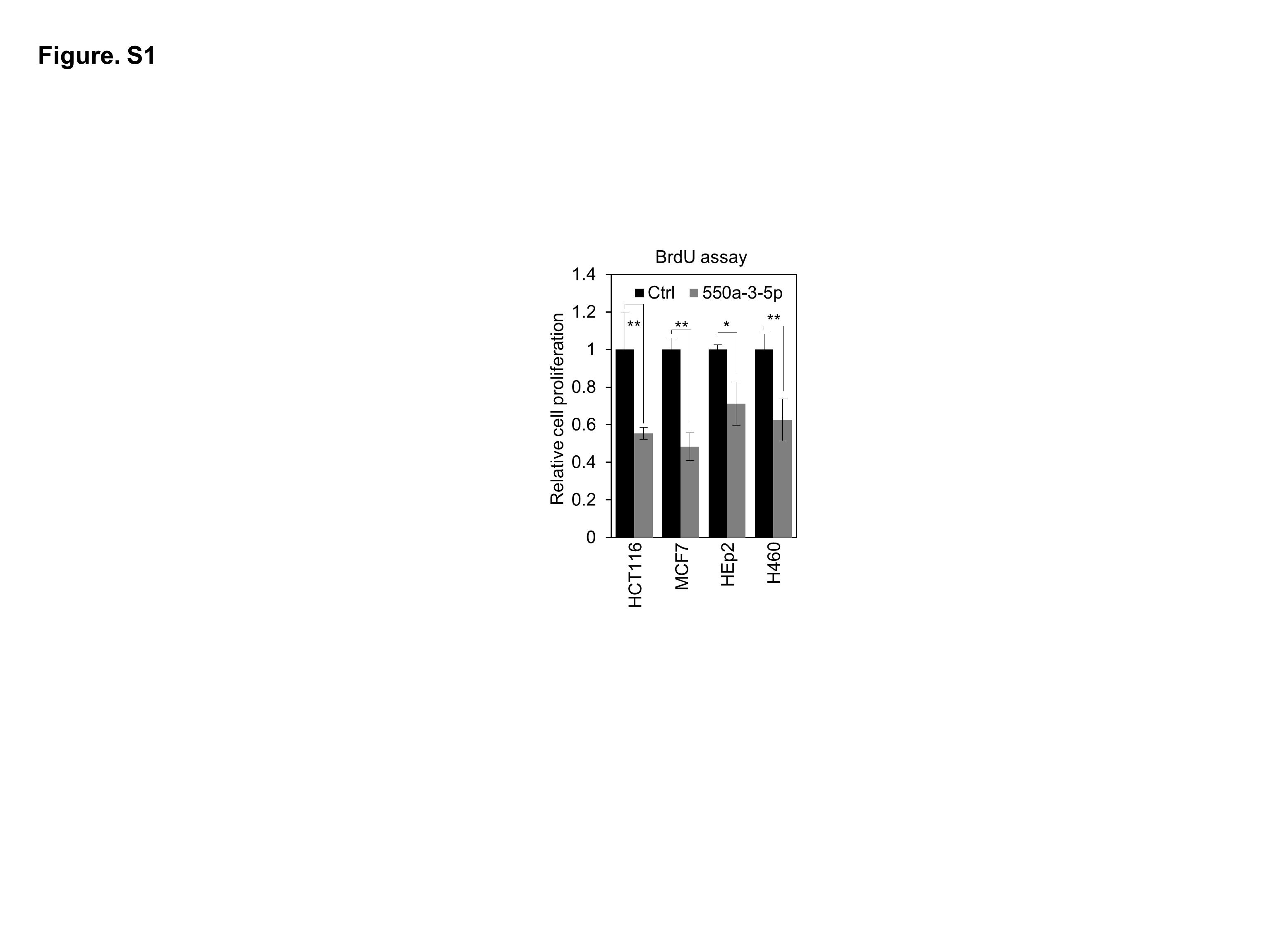

Supplement: Supplementary file 1 — Supplementary Figure 1 [file 41419_2018_698_MOESM1_ESM.tif]

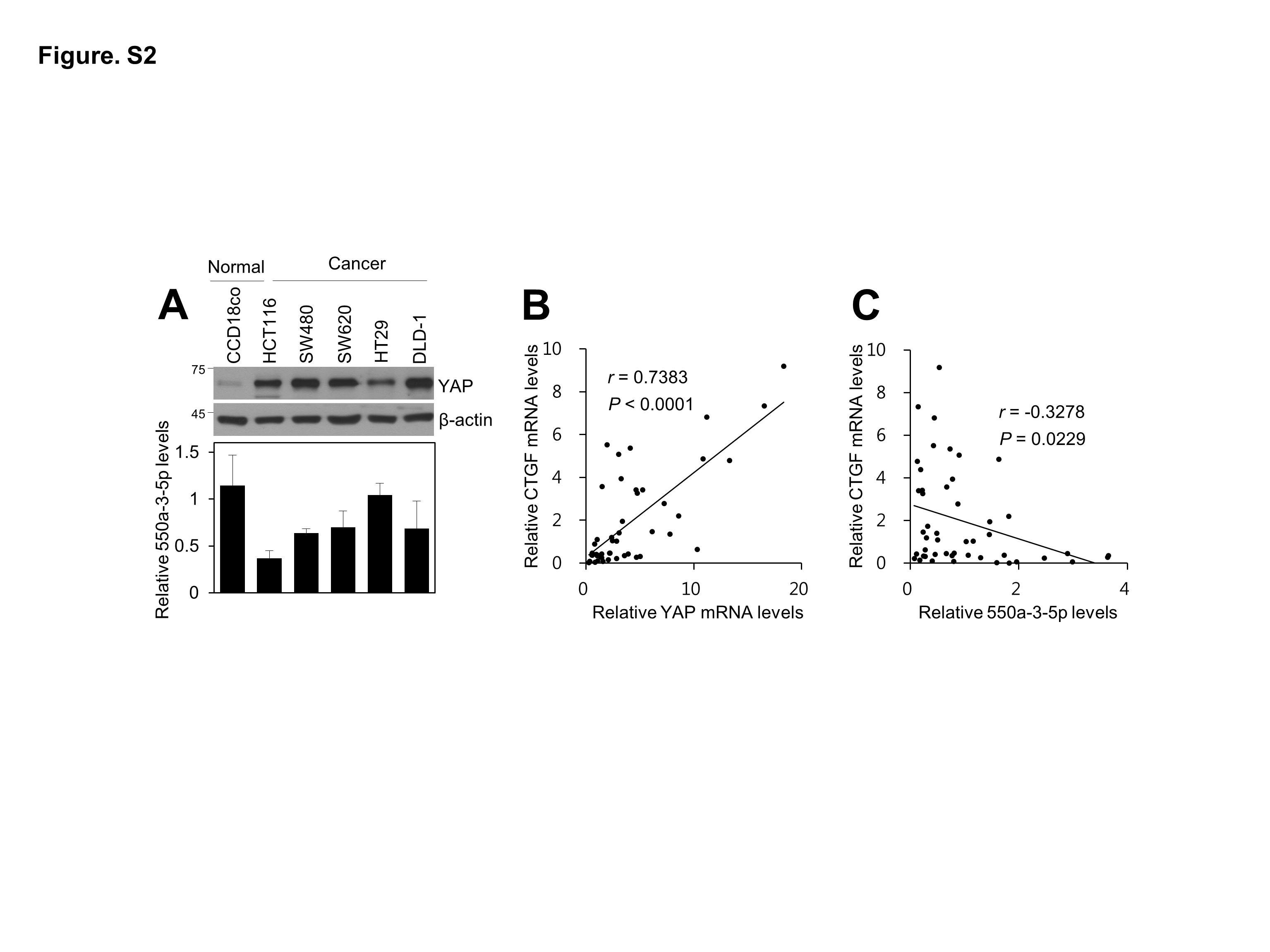

Supplement: Supplementary file 2 — Supplementary Figure 2 [file 41419_2018_698_MOESM2_ESM.tif]

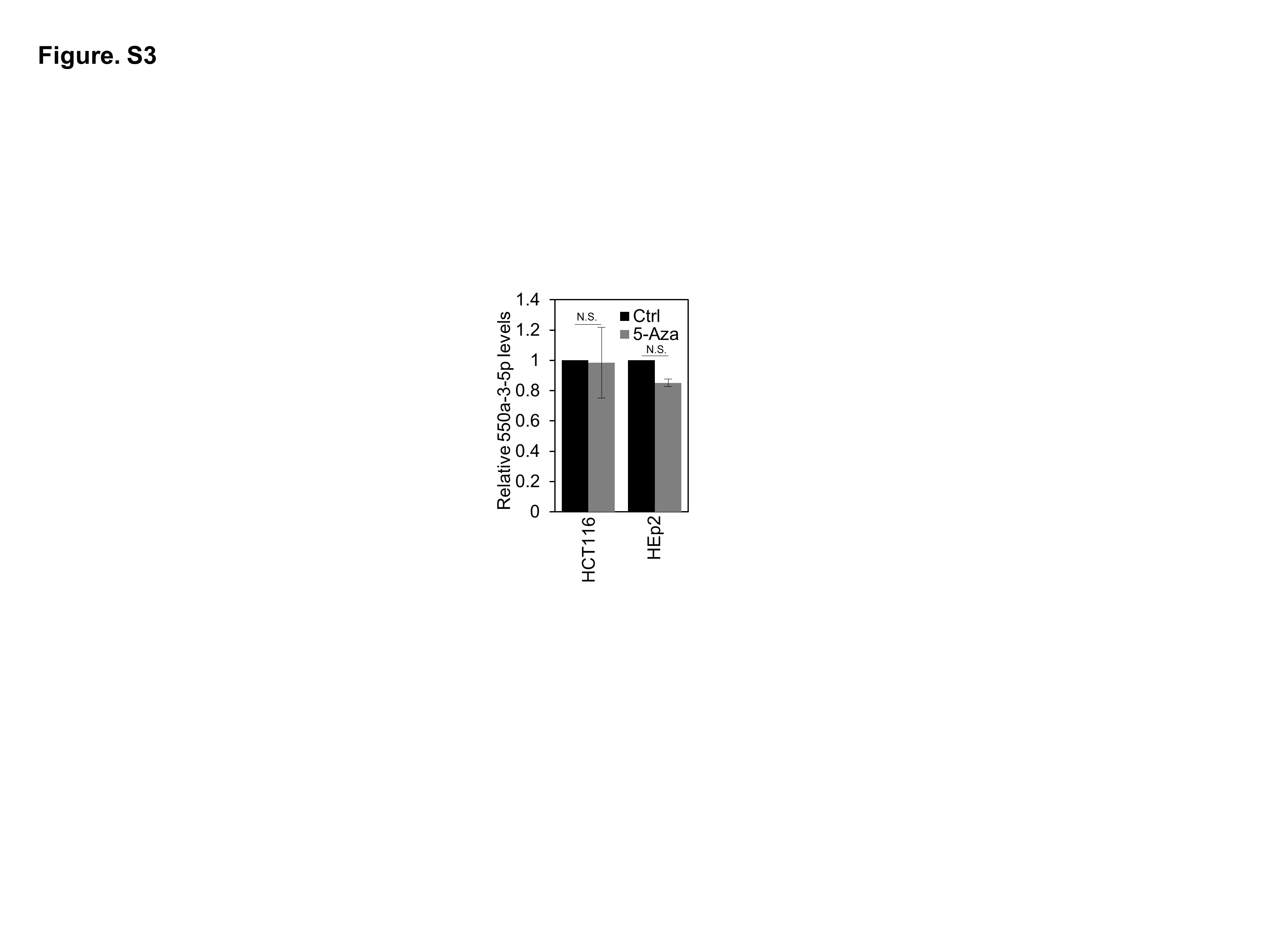

Supplement: Supplementary file 3 — Supplementary Figure 3 [file 41419_2018_698_MOESM3_ESM.tif]

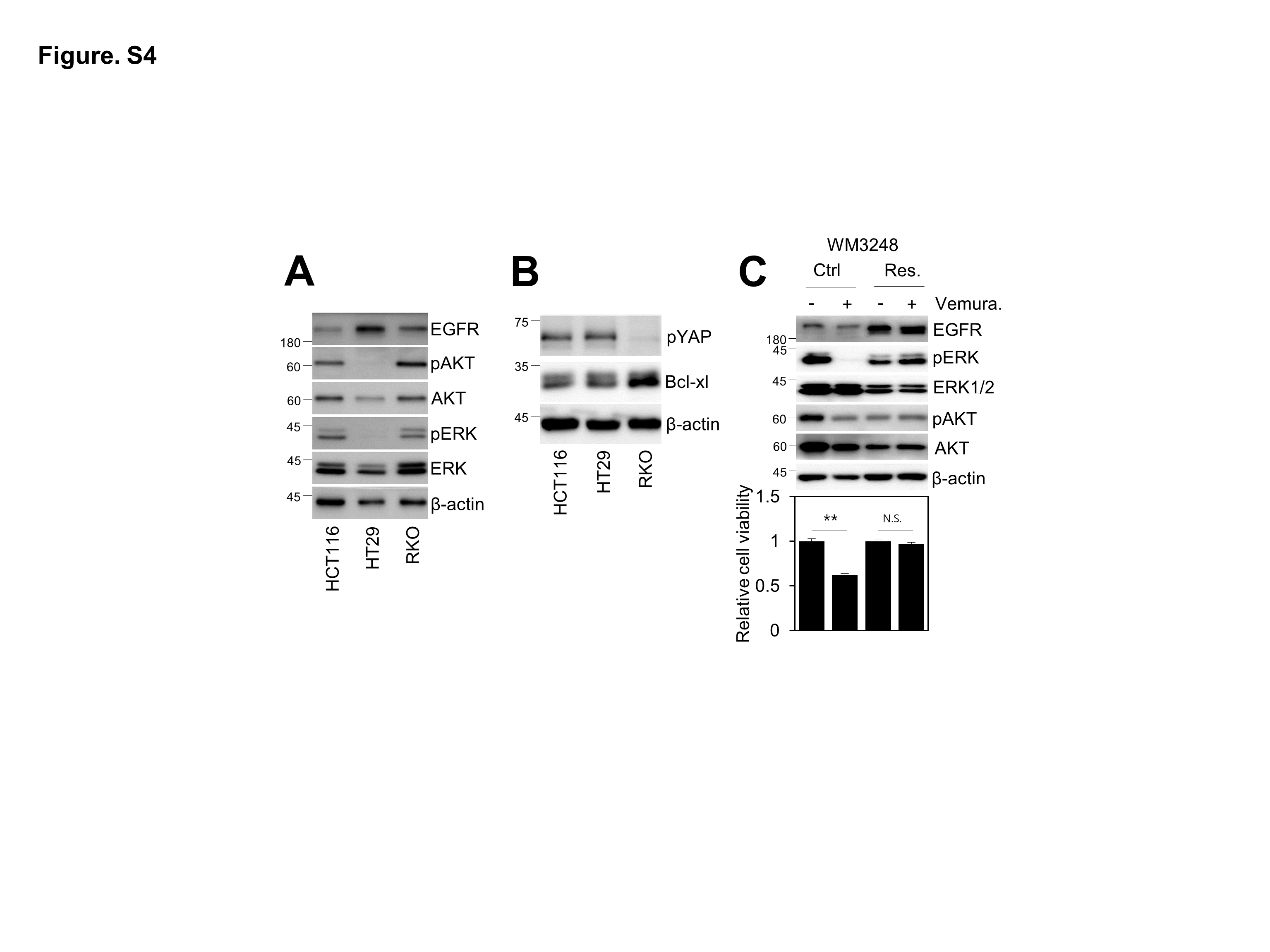

Supplement: Supplementary file 4 — Supplementary Figure 4 [file 41419_2018_698_MOESM4_ESM.tif]

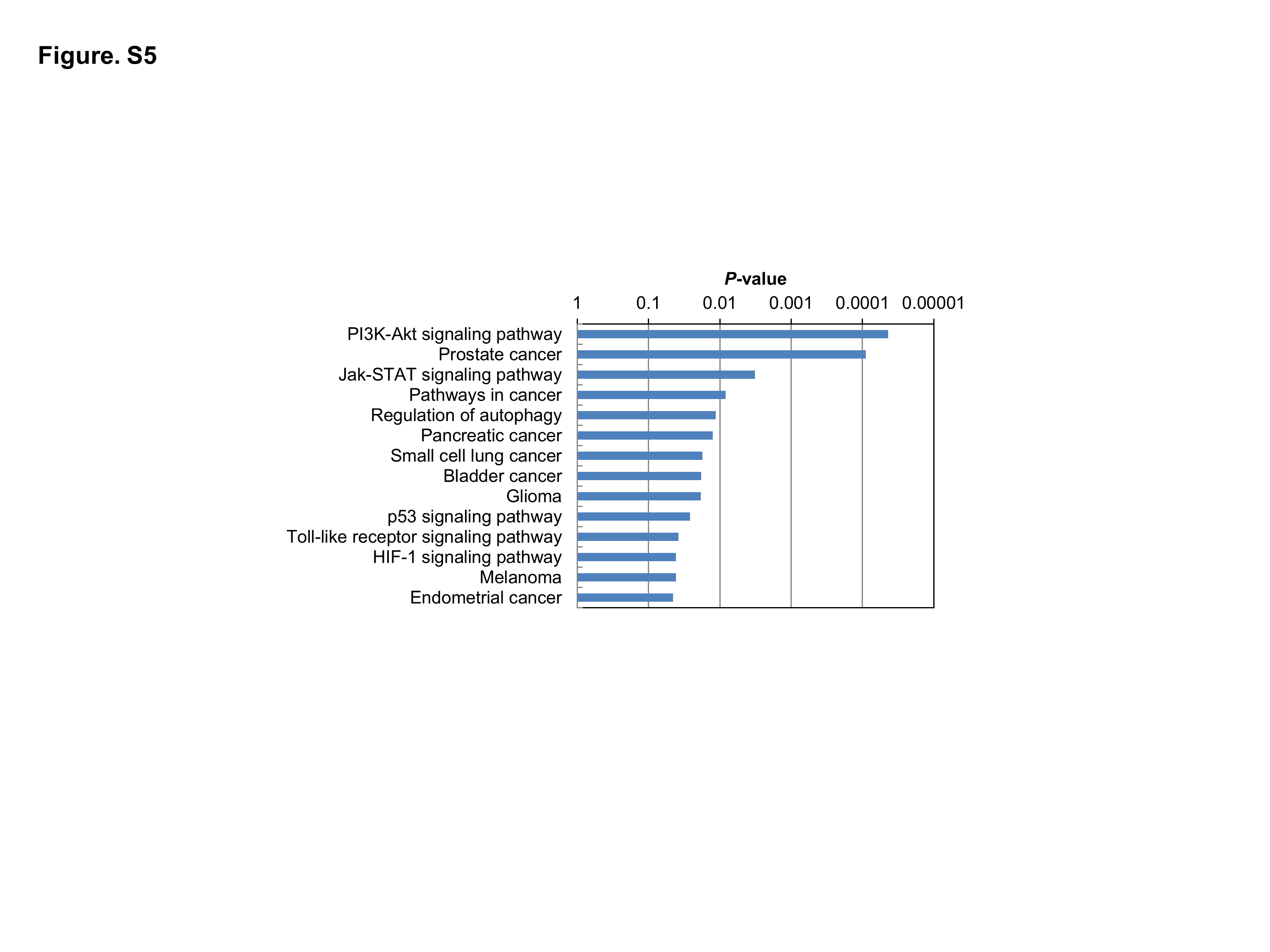

Supplement: Supplementary file 5 — Supplementary Figure 5 [file 41419_2018_698_MOESM5_ESM.tif]
