# Supplementary material for: miR-550a-3-5p acts as a tumor suppressor and reverses BRAF inhibitor resistance through the direct targeting of YAP
Source: Cell Death Dis. 2018 May 29;9(6):640. doi: 10.1038/s41419-018-0698-3 (PMC5974323; doi:10.1038/s41419-018-0698-3)
Supplement: Supplementary file 6 — Supplementary figure legends [file 41419_2018_698_MOESM6_ESM.docx]

**Supplementary Figure Legends**

**Fig. S1.** miR-550a-3-5p inhibits cell proliferation in various cancer cell lines. HCT116, MCF7, HEp-2, and H460 cells were transfected with either control miRNA or miR-550a-3-5p for 48 h and cell proliferation was determined by BrdU assay. The data are presented as the mean ± standard deviation of three independent experiments; *P* < 0.01 (**) and *P* < 0.05 (*).

**Fig. S2.** Inverse correlation between YAP signature and miR-550a-3-5p in colon cancer cells and tissues. **a** YAP or miR-550a-3-5p expression was analyzed by immunoblotting with YAP antibody (upper panel) or qRT-PCR (lower panel), respectively. β-Actin (upper panel) or U6 (lower panel) was used as a loading control, respectively. **b** Positive correlation between YAP mRNA and CTGF mRNA in colon cancer tissues. **c** Inverse correlation between CTGF mRNA and miR-550a-3-5p in colon cancer tissues. GAPDH or U6 were used as the loading controls, respectively (**b** and **c**). The data represent typical results and are presented as the mean ± standard deviation of three independent experiments.

**Fig. S3.** Non-epigenetic regulation of miR-550a-3-5p. HCT116 and HEp-2 cells were treated with 50 μM 5-aza-2-deoxycytidine for 72 h. miR-550a-3-5p expression was analyzed by qRT-PCR. U6 was used as a loading control. The data are presented as the mean ± standard deviation of three independent experiments. N.S.: not significant.

**Fig. S4.** Analysis of BRAF-inhibitor resistant colon cancer and melanoma cells. **a**, **b** HCT116, HT29, and RKO cells were analyzed by immunoblotting with the indicated antibodies. **c** Parental and vemurafenib-resistant WM3248 cells were treated with 2 μM vemurafenib or DMSO for 24 h. The cells were analyzed by immunoblotting with the indicated antibodies (**c**, upper panel). The cell viabilities were determined by WST-8 assay (**c**, lower panel). The data represent typical results and are presented as the mean ± standard deviation of three independent experiments. N.S.: not significant.

**Fig. S5.** The predicted pathway of miR-550a-3-5p. The putative pathway of miR-550a-3-5p was predicted by the DIANA tool. Several pathways that are putatively regulated by miR-550a-3-5p are listed in order of significance, according to p-value.
